# Managing courts in competitive authoritarian regimes: Co-optation, repression and resistance in Hungary

**DOI:** 10.1007/s12286-024-00621-y

**Published:** 2025-01-14

**Authors:** Etienne Hanelt, Attila Vincze

**Affiliations:** 1 Judicial Studies Institute, Masaryk University, Veveří 70, 611 80 Brno, Czech Republic; 2 Centre for Socio-Legal Studies, University of Oxford, Manor Road Building, Oxford, OX1 3UQ UK

**Keywords:** Competitive authoritarian regime, Courts, Co-optation, Hungary, Repression, Rule of Law, Gerichtsbarkeit, Kompetitive autoritäre Systeme, Kooptation, Rechtsstaat, Repression, Ungarn

## Abstract

More than a decade of democratic backsliding has turned Hungary into a competitive authoritarian state. The government has initiated many judicial reforms to exert control over the judiciary, yet needed to maintain plausible deniability due to formal international and constitutional standards of judicial independence. Based on interviews with Hungarian judges and experts for the judicial system, we explore techniques of managing the courts. In particular we study mechanisms of co-optation and soft repression and explain why the resistance of judges was weak. We find that a complex web of informal patronal politics undermines judicial independence in practice. Moreover, the regime fosters competition between three clients who are entrusted to manage and control the judiciary: the heads of the Supreme Court (Kúria), the National Judicial Office, and the Constitutional Court. Their resources and power ebb and flow with their reliability and performance. We conclude that competitive authoritarian regimes can maintain formally independent judicial institutions by delegating and incentivizing control over the judiciary. They thereby escape measurement, maintain plausible deniability, and evade international pressure.

## Abstract (de)

Die allmähliche dekadenlange Autokratisierung hat Ungarn in einen kompetitiven autoritären Staat verwandelt. Dabei wurde die exekutive Kontrolle über die Justiz durch gesetzgeberische Reformen ermöglicht. Die Änderungen weichen von den formalen Standards richterlicher Unabhängigkeit nur leicht ab, öffnen aber den Raum für informelle Institutionen und Praktiken, was der Regierung erlaubt, eine solche Kontrolle plausibel abzustreiten. Auf der Basis von Interviews mit ungarischen Richtern und Experten erforschen wir die Techniken, mit denen die Gerichte kontrolliert werden. Wir untersuchen Mechanismen der Kooptation und der weichen Repression, die Anreize für Konformismus schaffen, was auch den relativ kleinen Widerstand der Richterschaft erklärt. Im Ergebnis finden wir, dass das ungarische Regime die Kontrolle und das Management über die Justiz auf drei Institutionen aufgeteilt hat: den Obersten Gerichtshof, das Verfassungsgericht und das Nationale Justizamt. Diese drei werden auch gegeneinander ausgespielt: ihre Kompetenzen und Ressourcen steigen und fallen abhängig von ihrer Zuverlässigkeit und Effektivität. Ungarn, als ein paradigmatischer Fall des kompetitiven autoritären Staats, kann den Anschein richterlicher Unabhängigkeit dadurch bewahren, dass die Kontrolle über die Justiz indirekt durch Patronage-Netzwerke und Anreize erreicht wird. Dies verringert internationalen Druck und erschwert es, das Regime gegen Benchmarks zu messen.

## Introduction

Hungary is the first EU country that left the group of democracies (Freedom House 2020). Most commentators see Hungary as within the group of “hybrid regimes”, ranging from a “diffusely defective democracy” on the one end (Bogaards 2018) to a “prime example of a competitive authoritarian state” on the other (Way and Levitsky 2019). The European Parliament (2022) settled on the term “hybrid regime of electoral autocracy”. These definitions mirror that institutions of electoral democracy exist but are hollowed out. Diamond (2020) pointed out the role of a feeble and diminishing Rule of Law as an enabler of “democratic regression”, while Levitsky and Ziblatt (2019) discussed the role of “capturing the umpires” in the death of democracies. Thus, independent judiciaries are often among the first victims of backsliding states.

While Hungary has largely maintained *formally* independent judicial institutions, they are undermined in practice, putting the Rule of Law under pressure. We provide an account of how judicial independence was subjected to mechanisms of co-optation and “soft” repression in Hungary. This article adds to a corpus of literature that looks at how Viktor Orbán obtained control of the judiciary despite constitutional constraints and supranational scrutiny.

This article contributes to the literature in three ways. First, international studies mostly zero in on apex courts and especially Hungary’s Constitutional Court (Drinóczi and Bień-Kacała 2021; Pap 2018; Szente and Gárdos-Orosz 2018) or the Supreme Court (Vincze 2015; Kosař and Šipulová 2018) and reflect very rarely the regular judiciary. While this narrow focus of the court-packing literature (Kosař and Šipulová 2023) is understandable, it misses most of the judiciary and, with it, most of the cases. The apex courts work differently than the ordinary ones. They deal with high-profile cases. Constitutional courts, moreover, have a reduced focus on points of constitutionality only and a tighter control of their docket. Furthermore, fifteen constitutional judges can be more easily hand-picked than two-and-a-half-thousand rank-and-file judges, who deal with the bulk of the caseload. Because they cannot be controlled directly, some shrewdness is required. So, we look at the on-the-ground operation of the judicial system.

Secondly, we describe informal mechanisms and their interplay with formal institutions to control the judiciary, focusing on co-optation and soft repression (Gerschewski 2013). We provide an explanation of how Orbán succeeds in controlling the judiciary while avoiding adverse reactions from the international community, which can be important for regime stability (Levitsky and Way 2010). Specifically, we ask how a competitive authoritarian state manages the judiciary to deliver the expected results while maintaining a level of plausible deniability.

Thirdly, we rely on unique and novel data. Despite the scholarly interest in the Hungarian judiciary, interviews have rarely been used in the literature. A report by Amnesty International Hungary (2020) has used some interviews but largely limited sampling to existing contacts, which necessarily tilted the interviews towards rather regime-critical judges. Puleo and Coman (2024) relied on interviews with four Hungarian judges with the specific aim to understand (the lack of) resistance strategies as part of a comparative inquiry. Our broader pool of judges and set of questions allow us to add insights and nuance to existing accounts of judicial (in-)dependence in Hungary. In particular, we study how different elements of control and capture interact and intersect with each other to create favourable outcomes for the Fidesz government.

This article proceeds in the following way. In the next section, we situate our study in the literature and introduce our analytical framework. In Section 3, we briefly set out our methods and data collection. Section 4 provides the empirical account of control in the Hungarian judiciary. In Section 5, we discuss our findings and conclude.

## Courts under competitive authoritarianism

Hybrid regimes became most relevant after the end of the Cold War as many closed autocracies around the world broke down. This was famously hailed as “the third wave of democratization” (Huntington 1993). However, in the following years, it became clear that many of the countries got stuck during the regime transition. Levitsky and Way (2002, 2010) classified the resulting regime type as “competitive authoritarianism”, defined as “civilian regimes in which formal democratic institutions exist and are widely viewed as the primary means of gaining power, but in which incumbents’ abuse of the state places them at a significant advantage vis-à-vis their opponents. […] Competition is thus real but unfair” (Levitsky and Way 2010, p. 5). Hence, democratic institutions are more than a façade in these regimes, yet the playing field is systematically tilted, leading to an unfair advantage for the incumbent. We use the label of “competitive authoritarianism” following the regime classification scheme of Howard and Roessler (2006, p. 367) since elections in Hungary are contested, and while mostly free, they are not fair (ODIHR 2022; Scheppele 2022).

More recently, attention turned to the threat emanating from the deterioration, regression, backsliding, eroding or decaying of democracies. This phenomenon is driven by “executive aggrendizement” of elected governments (Bermeo 2016) and is “incremental in nature” (Haggard and Kaufman 2021, p. 28). Existing democratic institutions are undermined and hollowed out, yet persist in their general form. Lührmann and Lindberg (2019, p. 1104) have pointed to the “legal facade” that contemporary cases of backsliding states exhibit. Similarly, Scheppele (2018) described a method of “autocratic legalism”. Common to these accounts is the tacit acknowledgement of the backsliders’ need to escape international scrutiny, which they do by following abusive legal tactics.

Gerschewski (2013) described a framework of “three pillars of stability”. On this view, elites are co-opted, the opposition is repressed, and the regime creates legitimation narratives to prop up its popularity. The applicability of this framework for competitive authoritarian regimes was debated controversially. Lueders and Croissant (2014) found that it did not work for competitive authoritarian regimes, but Schneider and Maerz (2017) disagreed. This question can be left aside here, as our dependent variable is not regime stability. The scholarship differentiates further between “hard” and “soft” repression (Lueders and Croissant 2014, p. 332). “Hard” repression, i.e. violence, is not used by the Hungarian regime. On the other hand, “soft” repression, for example, through surveillance, and employment and benefits withdrawal are commonplace (Mares and Young 2019). Gerschewski (2013) described how both formal and informal methods of repression and co-optation are used by autocratic regimes.

Our article does not discuss how regimes survive. Instead, we are interested in the mechanisms of soft repression and co-optation in a competitive authoritarian regime that is “externally constrained” (Bozoki and Hegedűs 2018). For this, we provide a case study of Hungary’s judiciary. Key to backsliding in democracies is “capturing the umpires” and foremost the courts to remove judicial checks on executive and legislative power (Levitsky and Ziblatt 2019; Haggard and Kaufman 2021, p. 37). Captured courts can be used to legitimise reforms, enact otherwise politically costly changes under the guise of judicial independence, and punish opponents (Sadurski 2019).

The techniques such illiberal governments use vary. They attempt to take control of the appointment process to capture the centre of constitutional interpretation (Wyrzykowski and Ziółkowski 2021; Kosař and Šipulová 2023). To maintain the democratic façade, formally existing judicial independence is undermined by a complex web of informal institutions and practices (Kosař et al. 2023), which compete with formal institutions and turn them inside out (Helmke and Levitsky 2004; Lauth 2000). Thereby, the formal rules retain a façade of legality (Lauth 2017). Institutional changes help to understand the potential for undermining judicial independence in Hungary, but they do not adequately explain the functioning of the judiciary. Hence, understanding the formal judicial institutions and their reforms is necessary but insufficient to explain the functioning of the judiciary under competitive authoritarianism. With an onslaught of changes to all branches of Hungary’s judiciary and a continuous reconfiguration of the institutions and their powers vis-à-vis each other, we argue that it is necessary to look deeper at informal institutions and practices to manage the judiciary in the competitive authoritarian state (Lauth and Sehring 2009) and to reflect forms or lack of judicial resistance (Šipulová 2024).

Recent research has paid renewed attention to informal institutions in judicial bureaucracies (Kosař et al. 2023), and as part of Hungary’s backsliding and how it helped evade the EU (Zgut 2022). Informality within a tailor-made formal structure helps to manage judicial decision-making: controlling the incoming cases (“gatekeeping”), “channelling” sensitive cases to trustworthy judges, incentivizing behaviour with “carrots and sticks”. On top of that, “emergency breaks” against unfavoured decisions allow the government to obtain favourable outcomes (Vincze 2023a).

We analyse the workings of the Hungarian judicial system: how are judges co-opted and, if necessary, repressed into obedience and why does this work so well, meeting with little resistance? We argue that co-optation takes place through a multi-layered web of patronal politics (Hale 2014). Körösényi et al. (2020) identified this as a general attribute of the Orbán-regime. “Patronage” is a form of clientelism; the two terms are sometimes used interchangeably (Hicken 2011). Patronage is often used specifically for the use of state resources by the patron to gain support from the client and reward them (Hicken 2011; Stokes 2011). Demonstrations and anticipation of loyalty are the prerequisites to appointment or promotion to the top ranks of the judiciary. Underneath, a layer of court presidents does the government’s bidding on the local level, including the use of soft repression. The key players receive formal powers to foster and maintain the informal patronal networks.

Incentives are structured to reward loyal judges and judges who cooperate or stay out of the way. Recalcitrant judges, on the other hand, are subject to soft repression. While we have clear evidence of “red lines” as regards the intervention in judicial decisions, the use and abuse of administrative powers to punish obstreperous judges is common. Compared to their Polish and Romanian counterparts, Hungarian judges have not put up much resistance (Puleo and Coman 2024). For instance, Hungarian judges exceptionally make use of preliminary questions to reach out for the European Court of Justice to protect their independence (Vincze 2023b), a stark contrast to judges in Romania (Doroga and Bercea 2023). Judiciaries are bureaucratic environments, and the preferences internalised by judges shape their set of conceivable courses of action, which limits behavioural choice (González-Ocantos 2016). Most judges act according to the incentives they face. Because liberal-democratic norms have been rarely internalised and resistance is discouraged, partially via a legal ban on judges’ political activities, an environment was created where self-interested judges further the regime’s agenda, without the need for direct, top-down control.

## Methods and data

We describe and explain mechanisms of co-optation and soft repression under competitive authoritarianism, using a case study on Hungary’s judiciary. This case is paradigmatic for two reasons: first, judiciaries are typically among the first targets of backsliding governments; secondly, the Hungarian regime, while having become competitive authoritarian, is “externally constrained” due to its integration in the EU and Council of Europe (Bozoki and Hegedűs 2018). It is thus under extensive supranational scrutiny, requiring more reliance on informal mechanisms. In comparison, Poland has remained at the level of a flawed democracy and not deteriorated to the level of hybrid regime.

A qualitative case study approach allows us an in-depth exploration of mechanisms (Gerring 2004). It comes, however, at the cost of a narrower scope. In particular, the uniquely deep integration of Hungary’s competitive authoritarian state in the European Union reduces the applicability of our findings to other cases.

This article relies on fifteen semi-structured interviews that were conducted from June 2022 until June 2023. The interview guide was created as part of a multinational research project on informal judicial institutions and contains questions on informality within the judiciary, between judges and external actors and the extra-curricular activities of judges. The interview guide was not amended during data collection but participants were asked additional individual questions reflecting their position or were confronted with publicly known facts and information obtained in earlier interviews to probe their response and triangulate information. We use only information in this article which were cross-validated by at least two independent interviews. We tried to use the most readable quotations for a given piece of information.

Most interviews were conducted in Hungarian and online via video calling software. They were recorded, transcribed and translated to English and analysed to probe the mechanisms of co-optation and soft repression of Hungarian judges and provide evidence of why judges have not resisted these measures.

Ten active and former judges from different instances and five experts with inside knowledge of the judiciary (former politicians, researchers, university lecturers or attorneys) participated. We felt it necessary to combine the view from within the judiciary with those who work with the judiciary from the outside in order to filter out possible biases and motivated reasoning. We used different sampling methods including quota sampling (different tiers, regions, gender), convenience sampling (who is willing to talk), purposive sampling (of visible actors) and snowball sampling through chain-referrals (Tansey 2007, p. 769–770) to overcome difficulties recruiting participants faced by others (Puleo and Coman 2024, p. 52).

The lowest response rate was in case of judges in managerial functions and in case of (former) politicians (both 20%). Rank and file judges responded at a high rate of 91%. We tried to reach out to judges who had earlier discussed the judiciary—positively or negatively—publicly, because we assumed a higher willingness to talk. These judges were asked for further contacts in different regional areas or at different tiers of the judiciary. These attempts of snowballing were in many cases unsuccessful and promised contacts were not delivered or they did not respond to follow-ups. Taking these numbers into account, the response rate falls below 50%.

During the data collection, efforts were made to reach out to judges with different political and professional attitudes, however, it is unlikely that the sample reflects the full range of views, and might be slightly skewed towards critical perspectives. Nonetheless, it includes persons whose position has evolved from loyalist to critical of the government and also those who are supportive of the regime. While we do not claim representativeness for our sample, it reflects diversity vertically (tiers of courts), horizontally (regions), as well as in terms of gender composition. We pseudonymised the interviews and made extra efforts to protect the participants. This is a critical duty when conducting research in non-democracies (Loyle 2016, p. 933) and was an ongoing issue before and during the interviews for fear of retaliation. Therefore, we do not disclose further information about the court at which participants work or the exact date of the interview. For the same reason, interviewees were not approached through their official email addresses but through other means. We have divided participants into two categories: judges (J), the insiders, and experts (E), outsiders with intimate knowledge about the judiciary.

## Managing courts

Our case study is Hungary, a post-communist country that joined the European Union in 2004 and was then regarded as a democratic “frontrunner” in the region (Freedom House 2020, p. 2) regardless of the fact that the judiciary had many severe shortcomings (Fleck 2008; 2011), similar to other Central European countries (Kosař 2016). Since Viktor Orbán came to power in 2010, his government has radically changed the institutional setup of the country and many levers were pulled to bring the Constitutional Court, the Supreme Court, and the ordinary judiciary under control. This happened by organisational and personnel changes. Tools such as special remedies were created to enable control over the judiciary, and persons who were appointed were expected to make the best use of these tools (Vincze 2023a). Although the judiciary was thoroughly reorganised in 2011–12, the “reforms” have never stopped. Therefore, a particular feature of these changes is their fluidity: the system has been continuously reconfigured and adapted to circumstances, a strategy aptly described as “constitutional tinkering” (Körösényi et al. 2020, ch. 4).

The Hungarian judiciary consists of 139 courts in a four-tier hierarchy: 113 district courts (*járásbíróság*), 20 regional courts (*törvényszék*), 5 upper courts of appeal (*ítélőtábla*) and the Supreme Court (*Kúria*). The district courts are managed by the regional courts’ presidents, who have considerable power over the judiciary. The upper courts of appeal are the last instance appeal courts. The Kúria decides, based on extraordinary remedies, on points of law and formally serves the consistent application of law. In this latter respect, it has the power to issue binding interpretations of law, which was broadened in 2019. The Constitutional Court—among others—decides on the constitutionality of individual judgments based on extraordinary remedies called constitutional complaints since 2012. This enabled it to directly influence, correct and manage judicial decision-making, and therefore it also serves as an apex court. The judicial infrastructure has been managed by the National Judicial Office (NJO) since 2012. Its powers on personnel management (appointments, promotions, etc.) gave its President enormous influence.

One of the first targets of government attacks was the Hungarian Constitutional Court. Similarly to other illiberal cases, it was first defied and became a tool of oppression after its successful capture (Wyrzykowski and Ziółkowski 2021, p. 519). At first, its powers in fiscal matters were drastically curbed, the “*actio popularis*” was abolished and the appointment procedure for judges and that of the Court’s President was amended to give the Fidesz majority more leeway (Kovács and Tóth 2011, pp. 192–194; Kovács and Scheppele 2018, p. 191) and enabling it to gradually capture the court (Szente 2016; Kovács and Scheppele 2018). Once this was achieved, the Court’s powers were broadened and thanks to extraordinary remedies, it became a tool for controlling the judiciary.

The government renamed the Supreme Court to “Kúria” and dismissed the sitting President and Vice-President of the Court. While this was found to be contrary to the ECHR, it did not affect the mandate of the new President, Péter Darák (Vincze 2015, p. 448; Kosař and Šipulová 2018). A reform of the judiciary in 2019 widened the powers of the Chief Justice to micromanage the docket of the Kúria, and—by enacting a personified amendment—enabled the selection of a trusted person (András Varga Zs.) from outside the ordinary judiciary to exercise those powers (Kovács 2022).

Besides these early and quick attacks on the two apex courts of Hungary, the government also tried to bring the judiciary-at-large under control. Its principal method was the introduction of the NJO with a President who received vast powers of judicial administration. Its first President, Tünde Handó, had far-reaching influence on appointing, promoting, demoting, and disciplining judges. The forced retirement of judges in 2012 emptied one-tenth of all judicial positions and the vast majority of senior positions in the judiciary to which younger judges could be appointed or promoted (Halmai 2017, 471). These personnel changes, from which around one-fifth of the judiciary benefited, were orchestrated by the NJO. Although this was found to violate EU law, the new appointments have not been questioned by the EU. In the remainder of this section, we discuss the co-optation and soft repression of the Hungarian judiciary as well as the lack of resistance by Hungarian judges.

### Co-optation: loyalty, trust, incentives

Deep personal trust was the key component for the appointment of the three top actors in the Hungarian judiciary: the Presidents of the NJO, the Constitutional Court and the Kúria. The first President of the NJO was described by a judge as somebody “really coming from the inner circle” (J05). “It was common knowledge that on Sunday afternoons Anikó Lévai [Viktor Orbán’s wife] and Tünde Handó got together, because they had been good friends since their university days, and Viktor [Orbán] was also there” (J09). Handó was expected to control the whole judicial system (J05), achieve big organisational reforms to water down judicial self-governance and centralise powers in her hands (J04). This also included the central training of law clerks, which, as one judge said, served to establish personal bonds with the future generation of judges (J08). The personnel changes were made at the same time the judiciary was reorganised (J04). Handó had privileged access to the government: “She could ask for anything—legislative changes, money—and she got it straight away” (J05).

Nonetheless, after her attempt to subdue the National Judicial Council—a judicial self-governing body with limited oversight over her—failed, she also clashed with the Association of Hungarian Judges (MABIE). These made her a target of international condemnation and thus a liability for the regime. Consequently, the political leadership’s trust in her faded away. Thus, her access to resources was cut back and she was demoted to be a judge at the Constitutional Court ahead of the expiration of her term of office (J05).

The new President of the Kúria was appointed in 2020 on the basis of loyalty. András Varga Zs. “was always very, very close to Fidesz” (J01). In order to make him eligible for the position, a special legislative amendment was necessary, making this “a very obvious political appointment” (J04). He “seems to be fulfilling a mission, and this mission is not about raising the Kúria to a higher professional standard, but about […] commanding decision-making at the Kúria” (J05). During a meeting of the National Council of the Judiciary, György Sennyey, the new President of the NJO, alleged, “many of us know that [he has] a pipe dream: to subdue justice. Anyone who expresses a contrary opinion is treated as an enemy” (NJC 2023). Varga Zs. once openly admitted that those who dominate the courts, dominate the law, and those who dominate the law have the power. For him, this struggle for domination was between the national and supranational judiciary (Varga 2022), reinforcing his known rejection of Strasbourg and the supranational case-law (J04).

This statement was said “to demonstrate his loyalty” towards the government (J05). He also got special powers by institutionalising a new extraordinary remedy, the “law uniformity complaint”. This enables Law Uniformity Chambers that are presided over by the President or the similarly trusted Vice-President to review and annul any decisions of the Kúria (J02, J05). This is described as his “brainchild” (J05), which he received to control the judiciary on a top-down basis (J05, E04). One judge also highlighted the connection between his appointment and the organisational change, namely the law uniformity complaint procedure enabling him originally to chair these special chambers and set them up, which was described as “unprecedented” (J04).

As the government’s attempt to control the whole judiciary via the NJO resulted in too many scandals, it realised that it suffices to control the apex courts (J05). As one judge put it:

“During the last five or ten years, there has been no change in the organisation, no change in the powers of the Kúria and the NJO, there has only been one change of person: [a] new President of the Kúria and a new President of the NJO. During the previous nine years, we had a very strong NJO and a grey Kúria. Now we have a very strong Kúria and a grey NJO” (J05).

A very similar trust and personal bond can be seen in the case of the new President of the Constitutional Court. The Court was dismantled after the landslide victory of Fidesz in 2010 (Vincze 2014) under the watch and passive assistance of Péter Paczolay, who was later rewarded with an ambassadorship in Italy and a judgeship in Strasbourg (J04). The institution was rebuilt after Tamás Sulyok was appointed as its President in 2015. He was earlier “a not really renowned farmer’s association lawyer” (E05) and became a judge of the Constitutional Court and later its President. Nonetheless, he was the then Minister of Justice’s former doctoral student and had a close connection to him. He was described as devoted to his former mentor, wanting to prove himself (E05). This he did: the whole Constitutional Court, during several years, “hardly ever made a ruling […] that was any surprise to the Minister of Justice, and none that was even unpleasant for the Minister of Justice” (E05).

Sulyok was known to allocate all the politically important cases to himself (J02). He proved his loyalty by backing the government in politically highly sensitive migration cases and introduced a limitation on the primacy of EU law supporting the aims of the government (Vincze 2022). Besides rubber-stamping governmental policy, the Constitutional Court had been redesigned to balance the Kúria’s power. For this purpose, a special constitutional complaint was established enabling public bodies to seek remedy against the final decisions of the Kúria (Vincze 2023a).

The politically appointed top actors maintain their own patronage networks of closely trusted persons. As one other judge put it: “there was an organisational change, centralization, and there was a personnel change, happening at the same time. And then that person appoints people who are loyal and faithful to him or her, or at least trustworthy people” (J04). In particular, the cooperation and assistance of the presidents of the regional courts (*törvényszék*) and the upper courts of appeal (*ítélőtábla*) are needed, because “the ideas of the National Judicial Office can be implemented through [them]” (J03). One judge called them the “prefects” of the NJO (J09).

Their appointment sometimes required active micro-management by the NJO, abusing administrative tools such as annulments of calls for application. This “rather sneaky solution” (E04) was controversial because it often promoted judges to important managerial positions without the support of a given court (J01, J04, J09). These interferences often discouraged others from applying again (Pálmai 2017). The active management of appointments also shows that the positions were used as rewards for loyal judges (J01, J04, J09), and enabled Handó to “occupy a significant part of the court management” (E04). As one judge put it, “the system is built on the over-performing middle managers” who “hoped for something from Tünde Handó” and were ready to support her (J10).

The forced retirement of the judges in 2012 happened smoothly, because of the collaboration of leading judicial figures who were later rewarded with managerial positions (J10). However, promotions need not be rewards: in some cases, they are “honeypots”. Judges working in sensitive areas like freedom of information or freedom of the press were offered lucrative positions to higher instance courts to get them out of the way. They were substituted with new ones who “have to learn the ropes anyway […] and that’s cool because a few dozen cases can be turned around in the meantime” (E04).

Court Presidents are rewarded if they cooperate with the President of the NJO: their courts get more resources and they receive better remuneration (J08) and considerable bonuses compared to rank-and-file judges (E04). Several judges reported that these court presidents were close allies and friends of the former President of the NJO (J09, J02, J04). The new one has no such personal network, but it is claimed that “it doesn’t matter that Tünde Handó is a constitutional justice, she continues to pull the strings very skillfully from the background” (J09, similarly J04).

Several judges describe the organisational structure by feudalistic analogies (J06, J05) and highlight the role of personal trust between the former President of the NJO, Tünde Handó, and a close circle of her friends (J08, J07, J09, J01). One judge spoke of “oaths of allegiance” (J09) necessary to obtain managerial positions. On one occasion, Handó explained whom she prefers to select for a job: “My personal principle is that I prefer to entrust the community [of the judges] for six years to those who have already done and proven themselves without a position” (Pálmai 2017). The person mentioned in that sentence was promoted from a first instance court to become the President of an Upper Court of Appeal under unusual circumstances (J04). Other persons had similarly successful careers under Hando’s management. As one expert explains, “the judicial leadership has been very carefully filtered over the last ten years. Virtually all the presidents of the regional courts (*törvényszék*), and now also the most important district court presidents, are elected from this [trustworthy] circle” (E04).

### Soft repression: the abuses of administrative measures

If co-optation is the “carrot”, soft repression is the “stick”. Wielding the stick is often left to the middle manager, who acted (and some claim they still act) as Handó’s proxies (J02). They were expected to do the politically sensitive work for the NJO President, such as keeping “problematic” judges under control. For example, the National Judicial Council became inoperable because its members resigned. Reportedly, “the local administrative leaders played a decisive role in this resignation, and they literally blackmailed, intimidated and threatened—not in writing, but verbally—the members of the National Judicial Council” (J09). Judges also claim that those court presidents receive protection from the President of the NJO. They face no consequences whatever scandal may arise (J01, J07, J09). One former judge explained: “Anyone who has not done this government a favour is worthless. And those who did favours should be rewarded. And those who went against it should be punished” (J04). The same was said about the new President of the Kúria: “He thinks only in terms of persons and he pays everybody off, so he pays off the bad and the good” (J05). When Varga Zs.’s appointment was proposed, the National Judicial Council—in its non-binding opinion—rejected his nomination.

He has obviously been fighting with the Judicial Council ever since the Judicial Council voted him down by 13 to one. The one who did not vote him down was Darák. Now [Darák’s] wife, Szilvia, is going to be a Kúria judge […]. So he [Varga Zs.] does not forget anything (J05).

The court presidents of the 20 regional courts and 5 upper courts of appeal, plus the President of the Kúria have a decisive influence on judges’ lives (J03, J07, J06, J09). Several judges confirm that there is a professional red line, that the court president must not directly intervene in adjudication itself (J03, J06, J09), but, as one put it,

in administrative matters, she has almost unlimited powers. The president has a very big role in terms of day-to-day work conditions, and […] the workload of a particular judge, the colleagues she works with, the workplace she gets, the computer equipment she gets, the quality of transcribers, whether she has an assistant or a clerk assisting her, or is allowed to work at home or not and have to be in the courthouse all the time (J03).

These decisions are discretionary, and there is no remedy against them (J03, J07, J06, J09). The judges,

receive bonuses at the end of the year, and these bonuses are discretionary and decided by the court presidents. And when the [National Judicial] Council wanted to check this and ask for data, the President of the Judicial Office rejected […] The Judicial Council is not able to get any data because […] they don’t have enough power and they don’t have enough support. […] It’s the system, it is really made not to supervise the politically appointed President of the National Judicial Office (J01).

One judge explained that she got different assignments and managerial duties (and with them a somewhat higher salary) as long as she remained loyal, but as soon as a conflict emerged between her and the President of the NJO, all these assignments were cancelled and she was harassed by different administrative measures, e.g. by never being granted deadline extensions (J09). While court presidents would not intervene in decision-making directly, their discretionary use of administrative tools is effective. It often happens that formerly granted benefits and allowances, such as teaching at a university or working from home are cut back in case of a conflict with the president (J06, J01, J05).

Interviewees explained the limits of this style of soft repression: if someone does not have any other ambition than to be a judge in the courtroom and does not need anything from the court president there are no formal ways to influence them, but if they do need something, the president can grant or refuse those allowances at will (J06) without giving reasons or fearing external control (J07). Not that the judges were keen on that control: “I do not see any evidence that judges have it in their guts to seek a kind of legal protection” (J03).

The tolerance for minor mistakes like not keeping deadlines and the use of disciplinary proceedings for infringements of duties very easily coincide with loyalty, sympathy and antipathy between the judge and court president (J09, J07). Personal relations are particularly crucial for promotion, which can be boosted or hampered by court presidents (J03, J07, J06, J09). Although there is an objective points-based system for promotions, many ways to obtain points such as study trips abroad, participation at conferences or obtaining further qualifications—require the approval of the court president (J09, J07).

Many judges concluded that if they are cooperative with the court presidents, they can get along (J08, J02), and if they are helpful they will be rewarded (J07). Judges are often driven by their career prospects (E03), and complaints or objections can set them back (J06, J08). Court presidents do not like conflicts and troublemakers at their courts (J06), and if judges are too active or recalcitrant, they can always be assigned one or two more complicated cases to keep them busy (J08). Some judges said that some court presidents dodge conflicts. If a judge is hard-nosed enough they sometimes rather give in (J02, J06). Yet court presidents find ways to retaliate later (J06).

The red line of not interfering directly in judgments has come under pressure since the appointment of the new President of the Kúria, who, according to interview sources, “doesn’t respect the rules of the game, the written and unwritten norms […] And that’s the trouble, that’s absolutely the way he thinks. He thinks like a politician. He doesn’t think like a judge” (J05). Two participants reported that he made a judge accountable for a decision (J05, J02). He reportedly yells at judges, “when there is something important that needs to be implemented and he could not manage to get [an important] case for himself” (J02), if he dislikes a decision (J05, J02) and if judges do not support a motion he puts forward (J05). Moreover, he is said to question the intellectual and professional capabilities of judges (J02) or their political neutrality (J05). His behaviour is no secret within the Hungarian judiciary (NJC 2023).

A participant reported that Varga Zs. took cases from judges in sensitive matters—like referendums—if they were not ready to write the expected decision (J02). If that does not work, judges report being pressured to sign decisions (J02) by withholding appointments and promotions or threatening them with assignments to other branches of law (from administrative law to civil law, for example; J02). The judges are rather perplexed about how to react. “There was no precedent for this in the past, and judges don’t [know how to fight], and that’s the great good fortune of this man [András Zs. Varga]. So there really is no opposition, and most likely there will not be any” (J05).

Some judges also explained the chilling effect of media campaigns. Hard-nosed judges who stand up for judicial independence often face attacks from media outlets going far beyond the limits of criticising the merits of a decision and depicting them as foreign agents. A judge describes this as “despicable”, “disgusting” and “emotionally very stressful” (J10). Another attributed these attacks to the judicial leadership and expressed concerns that these attacks undermine the authority of the judiciary as a whole (J09). Judges reported being disappointed in how the judicial leadership reacted to these campaigns and claimed that “the President of the Kúria even applauded it and basically said that [… they] deserve it” (J10).

### Internalised norms: explaining the lack of resistance

While co-optation and soft repression nudge judges to behave in certain ways, Hungarian judges often have internalised the norms of a non-independent judiciary in a competitive authoritarian state. The former President of MABIE, Lajos Makai, openly supported the forced retirement of judges in 2012 (Puleo and Coman 2024, p. 54). As a judge remarked, he was then later appointed by Handó as the president of the Upper Court of Appeal in Pécs, which was understood as a quid pro quo (J10). Other influential judges also discouraged judges from seeking remedy against forced retirement: “their duty is to work, not to run off to Strasbourg. So that was the message” (J10). Since judges did not resist the “most brutal, most drastic” method of forced retirement, milder steps did not meet resistance either (J10). Court presidents also remind judges of their duty of political neutrality, and that actions of resistance might be interpreted as forbidden political activity triggering disciplinary actions.

Despite abuses of administrative measures, judges often do not revolt even if they could but rather support and reelect their leaders:

there is also an element of self-colonisation, where a person whom we don’t like is reelected regularly by secret ballot. After an 80 percent result, I spoke to several people and I said, it is impossible that you did not vote for him, because the number could not have been that high otherwise. And then I hear that “there was no one else”, and “he would have been appointed anyway” (J06).

The helplessness is learned through painful lessons of soft repression. The temporarily appointed presidents received their promotion often against the judges’ will. After some time of cohabitation, they turned out to be able to “behave civilised” and “were no worse than the others” (E04). Moreover, these presidents enjoy the trust of the NJO and can bring resources to the court. As one observer remarked, nothing is more important than a few “new cars in the garage”, and judges understand that supporting the new president is also “good for them” (E04) at least in terms of material resources.

Moreover, court presidents can buy the loyalty of the most influential judges of the given court, who boost his or her good reputation by chatting with others and making sure that the president gets the right number of votes:

If you know that in six months, you will apply for another term as a president of the court, […] and […] there are five people with influence on the court – who are listened to, who can then influence the others – then you pay these five people [e.g. extraordinary bonuses] and then they will do what you expect from them, they will boost your good reputation and make sure that you get the right number of votes at the meeting where the judges give their opinion on your application (J07).

A majority of judges arrange themselves with their lukewarm situation, which was claimed to be “damn good for 90 percent” of judges (J06), who “don’t want anything more than to be left alone. 5 to 10 percent [of the judges] are outraged, and they are always pushed aside. If it were the other way around, it wouldn’t look like this” (J06). The same judge critically remarked that judges rationalise their passivity by referring to a “chilling effect”: “It’s not because of that, but, unfortunately, it’s because of something else. It is like a social embeddedness” (J06). The Hungarian Judicial Association was mocked by another judge for having had no further ambitions than organising sporting days, and it only found its new profile as guardians of the Rule of Law after Tünde Handó frontally attacked and humiliated its leadership (J10).

The apathy, passivity and lacking solidarity among judges was a recurring topic in the interviews (J01, J05, J08, J09, J10), which is an enabling factor for the political management of the courts. As one judge put it, their professional “immune system” is corrupted (J05), and they don’t know how to resist. The leadership depicts collective actions as banned political activities (J01, J09, J10) and, in doing so, highlights the risks of resistance.

## Discussion and conclusion

Despite the great interest in the capture of courts in Hungary, academic research thus far had overall, with few exceptions, neglected the ordinary judiciary and the subjective perspective of rank and file judges. By using interviews to collect novel data, we fill in the gaps in existing accounts of judicial capture and give nuance to the way the system works in practice.

Our conception of co-optation and soft repression in Hungary’s judiciary fills the gap in institutional accounts of Hungarian court reforms. While institutions are an important part of the story, they are also designed so that the government can plausibly deny control over the judiciary. Much-used indices such as V‑Dem are a case in point: while experts have rated the independence of the Constitutional Court as non-existent (0.87), the lower courts are said to be reasonably independent (2.5), with only a small deterioration (0.5) since 2010.^1^ Hence, detailed knowledge from the field is needed to understand the informal institutions and practices and their interaction with the formal institutions that are used for co-optation and soft repression of the judiciary.

Cynical accounts that imagine Viktor Orbán like a spider in the web pulling all the strings are mistaken too. Instead, our account of the Hungarian judiciary allows us to capture the nuances of managing the judiciary in a competitive authoritarian state. Our findings reject conceptions of the Hungarian judiciary as entirely top-down structured. First, direct interference in cases is rare and rarely necessary. The incentives, instead, are set up in a way to signal to judges the correct course of action. They are very aware of the incentives: the right decisions will be rewarded, the wrong ones punished.

Second, the leadership of the judiciary is not united. Instead, these clients face incentives to compete with each other for power and control of the Hungarian judiciary. Given that Orbán’s government is willing to continuously “tinker” (Körösényi et al. 2020) with the institutions, power and competencies flow between people and institutions in line with whether they fulfil the expectations set in them. Their power and influence can be cut back in favour of other clients if they do not respect the patron’s wishes (J04, E05). This institutional setting promotes competition between clients, who stand to profit from each other’s failings and minimises risk of delegation of power to the judiciary. As a respondent expressed it: “Our prime minister’s concept is to have two strong people in every area, preferably hating each other, and then it’s cool” (E05). It keeps clients on their toes as well: nobody is safe as anyone can be replaced.

Third, the system fosters initiative from the lower levels to gain the attention of the leadership, which may be rewarded. Hence, not every action is initiated from above and merely executed below. The busy and ambitious middle managers are key actors who often try to read between the lines to understand the expectations of the leadership and outperform them in the hope of being spotted and promoted (E05, J10). The same is true for some of the rank and file judges.

Fourth, there are instances where a principal-agent dilemma is visible. The agents put in place to manage the judiciary sometimes work their own agenda and pursue their own ambitions. Tünde Handó, who was described as a self-motivated person rather than a pure opportunist, was for long fighting against the introduction of separate administrative courts, “because that would have reduced her power and influence” besides weakening the judiciary as a whole (E04). Her personal and professional interests were in this case not fully in line with that of the government that appointed her.

While Hungary is a case that has attracted a lot of interest internationally, many similar abuses of administrative powers have been described in other Central European judiciaries (Kosař 2016; Blisa and Kosař 2018; Kosař and Spáč 2021). After the forced retirement of judges and the dismissal of the Supreme Court’s President in 2011/12, brute force has become less common and “soft” measures were predominantly used. This raises the question of why these sufficed and judges did not resist more, as they have in Poland and Romania (Doroga and Bercea 2023; Matthes 2022; Puleo and Coman 2024) or Israel (Gidron 2023), and have not reached out to international judicial fora either (Vincze 2023b). After some of them were punished for doing so, the others took notice and adapted their behaviour, learned and internalised the rules of the game (J04, J08, J10, E04). Judges’ conformity has developed due to a preference for individual gains in terms of career and working conditions over collective ones such as judicial independence or the Rule of Law, and was fostered further by lacking solidarity (J01, J05, J08, J09, J10) and their inability and unwillingness to act collectively (J05). By offering career and promotion for loyal or unproblematic judges, and suppressing individually minded ones, court management is capable of minimising resistance (E04).

Finally, this study has direct policy implications. Informal institutions and practices contribute as much if not more to putting the *Rechtsstaat under pressure* as legal changes. Yet, “the EU operates in a deeply legalized environment” and often disregards information about informal institutions when deciding whether to sanction backsliding governments (Zgut 2022, p. 308). This risks missing the lack of independence of the judiciary on the ground and could lead to failure in the implementation of policy, such as decisions on whether to withhold funds from Hungary under the Budget Conditionality Regulation in exchange for judicial reforms. Without intricate country knowledge, policymakers are at risk of over-estimating how independent judges actually are. Hungary has become a competitive authoritarian regime, after all.

## Footnotes

1 V‑Dem v14. The reported numbers are from 2023 and from the variables “High Court Indepedence” and “Lower Court Independence”, both on the original scale (_osp) that ranges from 0 to 4.
